# Influence of Polyols on the In Vitro Biodegradation and Bioactivity of 58S Bioactive Sol–Gel Coatings on AZ31B Magnesium Alloys

**DOI:** 10.3390/polym15051273

**Published:** 2023-03-02

**Authors:** Ashok Raja Chandrasekar, Emilia Merino, Amirhossein Pakseresht, Dusan Galusek, Alicia Duran, Yolanda Castro

**Affiliations:** 1 Centre for Functional and Surface-Functionalized Glass, Alexander Dubček University of Trenčín, 911 50 Trenčín, Slovakia; 2 Instituto de Cerámica y Vidrio (CSIC), Campus de Cantoblanco, 28049 Madrid, Spain; 3 Joint Glass Centre of the IIC SAS, TnUAD and FChPT STU, 911 50 Trenčín, Slovakia

**Keywords:** 58S sol-gel, magnesium alloy, bioactive glasses, polyols, biodegradable implants

## Abstract

The mechanical qualities of AZ31B magnesium alloys make them a promising material for biodegradable metallic implants. However, rapid degradation limits the application of these alloys. In the present study, 58S bioactive glasses were synthesized using the sol-gel method and several polyols such as glycerol, ethylene glycol, and polyethylene glycol, were used to improve the sol stability and to control the degradation of AZ31B. The synthesized bioactive sols were dip-coated onto AZ31B substrates and then, characterized by various techniques such as scanning electron microscopy (SEM), X-ray diffraction (XRD) and electrochemical techniques (potentiodynamic and electrochemical impedance spectroscopy), among them. FTIR analysis confirmed the formation of a silica, calcium, and phosphate system and the XRD the amorphous nature of the 58S bioactive coatings obtained by sol-gel. The contact angle measurements confirmed that all the coatings were hydrophilic. The biodegradability response under physiological conditions (Hank’s solution) was investigated for all the 58S bioactive glass coatings, observing a different behaviour depending on the polyols incorporated. Thus, for 58S PEG coating, an efficient control of the release of H2 gas was observed, and showing a pH control between 7.6 and 7.8 during all the tests. A marked apatite precipitation was also observed on the surface of the 58S PEG coating after the immersion test. Thus, the 58S PEG sol-gel coating is considered a promising alternative for biodegradable magnesium alloy-based medical implants.

## 1. Introduction

In the year 2022, the value of the global market for bio-implants was projected to amount to USD 117 billion. When looking into the future, it is anticipated that the market will reach USD 189.0 billion by the year 2028, expanding at a compound yearly growth rate (CAGR) of 8.12% from 2023 to 2028 [1]. Countless individuals suffer bone loss annually related to aging, accidents, congenital, or lifestyle-related diseases. Without surgery, the bulk of bone loss cannot be treated. Metals such as titanium or stainless steel in the form of screws, plates, and rods are currently the most popular biomedical implants for these surgeries, and they are often removed between around 2 and 5 years after the bones have healed [2]. 

Biomedical implants can be made of metals, ceramics, polymers, or composites. They can also be made of hydrogels, nanofibers, thin films, and scaffolds [3,4]. Polymeric and composite materials can be made into both load-bearing and non-load bearing, non-degradable and degradable implants. On the other hand, most metallic and ceramic materials are used as load-bearing and non-degradable implants [5]. Polymeric implants are known for being inexpensive, chemically stable, easy to shape and reshape, and resistant to corrosion. Polymeric implants are widely used in various biomedical applications, including vascular grafts, implants, wound dressing, sutures, catheters, meshes, stents, ligament repair, tendon repair, and valves used in cardiac surgeries [6]. Stainless steel, cobalt chromium alloys, various titanium alloys, gold, silver, magnesium, and nitinol are non-biodegradable metallic implants, whereas polymeric implants made of polyethylene, polymethyl methacrylate, and polyurethane are biodegradable [7]. Ceramics like calcium phosphates, hydroxyapatite, titania, and zirconia are often combined with polymers to combine properties like biocompatibility, degradability, and mechanical strength [8]. The primary issue with metallic implants is that they have stronger mechanical properties than human bone, which may have a stress-shielding effect [9]. Ceramic, polymeric, and composite materials, on the other hand, have substantially lower mechanical strengths, which might cause implants to fail under load-bearing situations [8] Degradable metal implants are now being researched as a solution to this problem, with iron, zinc, and magnesium being promising candidates [9]. In particular, magnesium and its alloys have been considered suitable candidates, not only due to their biocompatibility and degradability properties but also because their Young’s modulus (41–45 GPa) is comparable to that of human cortical bone (3–5 GPa) [10,11,12]. Magnesium also contributes to the regulation of muscle, neuron functions, blood sugar levels, blood pressure and to the production of proteins, bones, and DNA [13]. In orthopedic surgery, biodegradable implants are useful because a second surgery is not required to remove the implanted components [14,15,16]. To achieve this benefit, the biodegradable implant material must be biocompatible, biodegradable, release no hazardous by-products, and possess exceptional mechanical strength until the host bone recovers [2,16,17,18,19,20,21]. 

Nevertheless, in clinical application magnesium under physiological conditions suffers fast degradation. It degrades rapidly, leading to rapid production of hydrogen gas bubbles between the bone–implant interface and causing inflammation and pain, and restricting the use of magnesium as a biodegradable implant material [22,23]. To control the rapid degradation of magnesium, alloying elements such as aluminum, zinc, and rare earth elements were introduced to obtain magnesium alloys [24,25,26]. AZ31B is the most investigated magnesium alloy due to its capacity to resist rapid degradation under physiological conditions and low cytotoxicity [27]. Another approach to control the degradation rate is by applying surface modification techniques [28]. Several methods have been proposed, including anodization [27], plasma electrolytic oxidation [29], and the deposition of corrosion-resistant materials and/or bioactive coatings using the sol-gel method [30,31,32,33,34]. The sol-gel technique is an outstanding and low-cost technique for the preparation and deposition of biocompatible coatings because it not only inhibits the rapid degradation of the material, but also promotes bioactivity and therefore the bone regeneration [35,36].

Sol–gel derived biomaterials have been investigated for different applications, including immobilization of biologically active compounds, dental implants, and bone tissue regeneration. Bioactive glasses and silicates can be synthesized by sol-gel or melt-quenching processes [33,34]. However, glasses and silicates based on sol-gel technology are more desirable due to their amorphous nature and microporous structure [37,38]. In a recent study by Müller et al., they describe three new sol-gel deposited coatings, S85, S75, and S58, which were developed using the sol-gel process and deposited using the electrospray deposition [39]. They report the possibility of obtaining a homogeneous amorphous coating, which is advantageous for increasing bioactivity using sol-gel prepared coatings. Similar to this, Omar et al. reported that 58S bioactive glasses were synthesized by sol–gel deposition on stainless steel AISI 316L by spray coating had superior bioactivity to traditional melt-derived glasses due to their increased homogeneity, surface area, and porosity [40]. In another report by the same group on the deposition of 58S and 68S sol-gel on AZ31 and AZ91 magnesium-based alloys, it was found that the corrosion resistance of sol-gel-produced bioactive glasses was improved under physiological conditions [37].

However, the preparation of sol-gel derived bioactive glass requires good control of the synthesis parameters together with an appropriate selection of the precursors and thermal treatment. Several reports suggested that the use of organic polymers during the silica polymerization produces changes in the network formation, affecting the structural properties and aging of silica sol [41,42,43]. In the study on sodium alginate/glycerol thin films by Giz et al. the importance of glycerol in relation to structural properties such as stress relaxation in thin films is presented. According to their report, glycerol as an additive acts as a lubricant by replacing the stress-inducing hydrogen bonds in polyelectrolyte structures with hydroxides [44]. Stefanescu et al. report the importance of the interaction of ethylene glycol with silica precursors such as TEOS in obtaining a non-porous silica matrix with a large surface area, which can be used in numerous areas of silica sol-gel application [45].

Catauro et al., reported the preparation of a PEG/SiO_2_ hybrid sol-gel coating on grade 4 titanium and observed improvement in biocompatibility and 3T3 cell proliferation as the result of PEG addition. PEG in the hybrid sol was also found to influence the hydrophilicity of the coating. Crack-free coatings were achieved at a high PEG concentration in the sol [46]. Such coatings can protect metal substrates against corrosion or, in the case of biodegradable metals such as magnesium and its alloys, they can control the degradation profile of the metals. Glycerol, ethylene glycol, and polyethylene glycol are examples of polyols with excellent bioavailability, biocompatibility, and biodegradability [47,48]. In a previous report, ethylene glycol was used as a binder for sol-gel coatings on magnesium alloys [35]. However, little attention has been paid to the sol-gel synthesis of polyol-based bioactive glass sols and their use in sol-gel derived coatings. Most studies have concentrated on hybrid silica sol coatings rather than bioactive glasses. Consequently, the deposition of polyol sol-gel bioactive glass sols can provide interesting insights for future bioactive glass coating applications in tissue engineering and biodegradable implants.

The objective of the present study is the preparation of 58S bioactive glass coatings on AZ31B Mg alloys by sol-gel method to control corrosion and to obtain bioactive coatings. The addition of different polyols such as glycerol, ethylene glycol, and polyethylene glycol with an average molecular weight of 200 g·mol^−1^ is considered to optimize the sol stability and obtain an appropriate silica–calcium–phosphate network. The hybrid organic-inorganic (polyol-Si-Ca-P) bioactive sols and their coating deposition on AZ31B alloys were systematically studied in terms of their structural, bioactive, and biodegradation characteristics. The prepared polyol-modified bioactive sol-gel coatings promoted the biodegradability and biocompatibility of AZ31B alloys under physiological conditions without cytotoxic effects.

## 2. Materials and Methods

### 2.1. Materials

Tetraethyl orthosilicate-TEOS (SiC_8_H_20_O_4_, 98%, Sigma-Aldrich) and triethyl phosphite-TEPI (C_6_H_15_O_3_P, 98%, Sigma-Aldrich) were purchased from Shanghai, China. Calcium-L-Lactate-Ca-L-Lac (C_6_H_10_CaO_6_, ≥ 98%, Sigma Aldrich) was obtained from St. Louis, MO, USA. Nitric acid (HNO_3_ 65%, Labkem), glycerol-GLY (C_3_H_8_O_3_, 99%, Labkem), and ethylene glycol-EG (C_2_H_6_O_2_, 99%, Labkem) were purchased from Barcelona, Spain. Polyethylene glycol 200-PEG (HO(C₂H₄O)⁷nH, Merck), Hank’s Balanced Salt solution (Merck), and methanol (CH₃OH, Merck) were purchased from Darmstadt, Germany. Sodium bicarbonate (NaHCO_3_, 99.7%, VWR chemicals) was purchased from Gillingham, UK. A commercially available AZ31B magnesium alloy with the weight composition of 10.1% Al, 0.3% Mn, 1.0% Zn, <0.5% Fe, and Mg balance was provided by Dugopa S.A, Madrid, Spain.

### 2.2. 58S Sol Preparation

The 58S glass (60 mol % SiO_2_: 36 mol % CaO: 4 mol % P_2_O_5_) was prepared by using respectively TEOS, TEPI, and Ca-L-Lac as the precursors of SiO_2_, P_2_O_5_, and CaO, respectively. The 58S sol was prepared in two steps. First, Ca-L-Lac was dissolved in methanol. Simultaneously, TEOS, TEPI, and a polyol (GLY, EG, or PEG) were mixed and stirred for 1 h. Then, acidulated water (1N HNO_3_) was added drop by drop to the TEOS/TEPI/polyol solution and maintained under stirring for 1 h. Subsequently, Ca-L-Lac/methanol solution was added slowly to the sol and stirred for 1 h. The molar ratio of TEOS, TEPI, Ca-L-Lac, methanol, water, and polyol was fixed to 1.0: 0.14: 0.6: 7.5: 2.4: 4.0 and the total oxide concentration was 204 g/L. A similar sol was prepared without the addition of polyol. The different sols were respectively labeled as 58S GLY, 58S EG, 58S PEG, and 58S WP for the sols obtained respectively with glycerol, ethylene glycol, polyethylene glycol 200, and without polyol, respectively.

### 2.3. Dip Coating Process

The 58S coatings with and without polyol were deposited on microscopic glass-slide and AZ31B substrates. Before dip coating, the glass slides were ultrasonically cleaned in ethanol and dried with air. The sheets of commercial AZ31B were cut into plates 2 × 2 cm^2^ for pH and hydrogen evolution tests and 5 × 2 cm^2^ for structural, contact angle, immersion, and electrochemical characterizations. The AZ31B substrates were polished with silicon carbide sheets ranging from 120 to 2500 grit, cleaned with ethanol, and then, dried with air. All samples were dip-coated with a withdrawal rate of 30 cm/min. Coated samples were initially dried at room temperature for 1 h and subsequently cured at 160 °C for 1 h. 

### 2.4. Characterisations of the 58S Sols and Coatings

#### 2.4.1. Ellipsometry Characterization 

The thickness and the refractive index of 58S coatings deposited on microscopic glass slides after curing at 160 °C/1 h were determined by spectroscopic ellipsometry (alpha SE, J.A. Woollam). The measurements were made in the wavelength range of 250–1000 nm and at the angle of incidence 65°. 

#### 2.4.2. X-ray Diffraction Studies

An analytical X’Pert PRO theta/theta diffractometer (Malvern Panalytical, Madrid, Spain) was used to measure the crystal structure of the coatings. A grazing angle of 0.5° and 2θ range of 10–80 with a step size of 0.05° and accumulation time of 20 s per step was used. Cu-Kα radiation (λ = 0.15418 nm) was used as the excitation source was used.

#### 2.4.3. FT-IR Analysis

The structural characterization of 58S sols with and without polyols was carried out by Fourier Transformed Spectroscopy (FTIR, Perkin Elmer Spectrum 100 spectrometer, PerkinElmer, INC, Madrid, Spain), using a spectrometer with an attenuated total reflectance accessory (ATR). FTIR spectra were measured with a resolution of 4 cm^−1^ and 8 scans for each measurement. 

#### 2.4.4. Contact Angle Measurements

A ‘Drop Shape Analysis System’ Kruss DSA 100 system (Kruss, Hamburg, Germany) was used to measure the water contact angle. 40 µL of Hank’s solution was dropped over the sample surface on three randomly selected areas and the average contact angle was measured.

#### 2.4.5. Hydrogen Evolution Studies

The release of hydrogen from uncoated and coated AZ31B samples was evaluated using the procedure described by Song et al. [49]. The purpose of this experiment was to measure the amount of hydrogen gas released by the samples in Hank’s solution. The sample was immersed in Hank´s solution and the displacement of the solution in a graduated burette was measured [49] The amount of volume displaced is directly proportional to the amount of hydrogen gas released. For the experiment, the surface of the samples exposed to Hank’s solution was fixed to 1 cm^2^ and 20 mL of Hank’s solution was used. The device was also placed in a thermostatic bath to maintain the temperature at 37 °C ± 1 °C. The measurement was repeated three times to verify the reproducibility.

#### 2.4.6. pH Evaluation

The bare and coated AZ31B samples were immersed in Hank’s solution Solution at 37 °C ± 1 °C for seven days. The pH was monitored every 24 h. Similar to hydrogen evolution, samples with an exposed surface sample of 1 cm^2^ and 20 mL of Hank’s solution were used. Samples were analysed in triplicate to verify the reproducibility.

#### 2.4.7. Immersion Studies in Hank’s Solution

Immersion tests were conducted for seven days to study the formation of an apatite layer on the surface of uncoated and coated AZ31B samples. The samples were immersed in Hank’s solution. On the third and seventh days, they were extracted and analyzed using Raman spectroscopy. Raman spectra were recorded using a confocal Raman microscope (WiTec, Ulm. Germany) with a spectral resolution of 0.02 cm^−1^ coupled with an AFM instrument (ALPHA 300RA, WiTec, Ulm, Germany) and with 532 nm excitation laser. The images were analyzed with the WiTec Project Plus 2.08 software.

#### 2.4.8. Electrochemical Characterization of the 58S-Coated Samples 

The degradation of AZ31B alloys and 58S coated alloys was investigated using DC and AC signals in a Gamry FAS2 electrochemical unit (Gamry, United Kingdom, Warminster). Electrochemical impedance spectroscopy (EIS) was also applied, using a saturated calomel electrode (SCE, Radiometer, Hach Lang GmbH, Germany, Düsseldorf) as the reference electrode, metal samples as the working electrode, and platinum wire as the counter electrode. Hank’s Balanced Salt Solution was used as the electrolyte and an area of 0.78 cm^2^ was exposed to the solution. The EIS was done in the frequency range from 105 Hz to 0.1 Hz with a sinusoidal amplitude of 10 mV rms AC voltage applied at the open circuit potential. Three measurements of each sample were performed, and the most representative measurement was plotted. Zview 2.0 programme was used to fit the impedance plots with a compatible equivalent circuit.

## 3. Results and Discussions

### 3.1. Characterization of 58S Sols without and with Different Polyols and Their Coating Deposition 

Transparent 58S sols were obtained without the addition of polyol and with the addition of different polyols; GLY, EG, and PEG. Table 1 summarizes the aging times of 58S GLY, 58S EG, 58S PEG, and 58S WP sols, determined as the day in which the sol turns into a gel. The 58S PEG sol had the lowest stability, at 3 days. This was associated with the polymer long-chain and high molecular weight of PEG compared to the other polyols. It also contains more hydroxyl (OH) groups than other polyols, promoting the hydrolysis reaction and the crosslinking between the PEG and TEOS hydrolyzed molecules, thus increasing the viscosity. The 58S and 58S GLY sols were stable for 4 days, and the 58 EG sol remained stable for a longer period of 5 days. 

**Figure 1:**
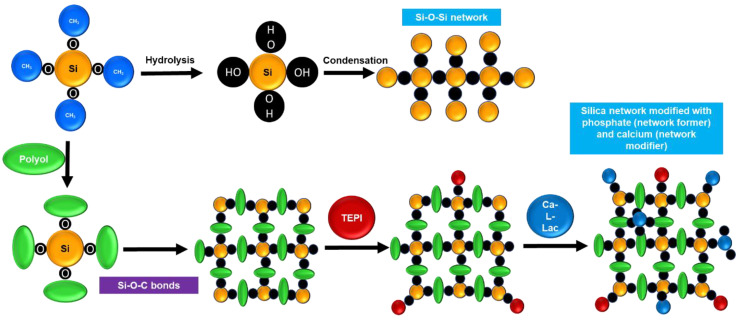
Schematics of conventional Si-O-Si network formation and influence of polyol, TEPI, and Calcium L-lactate in Silica network formation.

In general, the hydrolysis and polymerization reactions of TEOS take place in presence of water and under acidic/basic conditions to produce a crosslinked Si-O-Si network depending on the synthesis conditions. In our scenario, the presence of a polyol alters TEOS hydrolysis; as a result, hydrolyzed TEOS molecules (Si-O-) can interact with the polyol molecules (hydroxyl groups) and produce Si-O-C bridges, altering the formation of the Si-O-Si network as described by Ravaine et al. [43]. The degree of modification depends on polyol chain length and molecular weight and affects the sol stability together with the coating’s structural and biological properties [45]. Figure 1 depicts the possible reaction between the TEOS precursor and polyols. For coatings, the stability of the 58 EG sol is the only factor to consider; however, for a degradable implant application, the stability of the 58 EG sol is merely one of several factors to consider.

58S coatings were prepared using the 58S GLY, 58S EG, 58S PEG, and 58S WP sols by the dip-coating process at 30 cm/min and heat treatment at 160 °C/ 1 h. The thickness of the coatings was measured by ellipsometry, as reported in Table 1. In general, the thickness of the coating is affected by the addition of polyols, with the 58S PEG coating being the thickest, around 1.76 µm. Polyol was added, and the result was associated with the viscosity of the 58S PEG sol. The thickness of 58S WP and 58S EG coatings was similar, 1.5 µm, and the thickness of 58S GLY coating was 1.65 µm. In a similar study by Agustín-Sáenz et al. on mesoporous silica coatings, the authors report the increase in coating thickness with respect to the different surfactants used in their study [50].

### 3.2. X-ray Diffraction Studies

**Figure 2:**
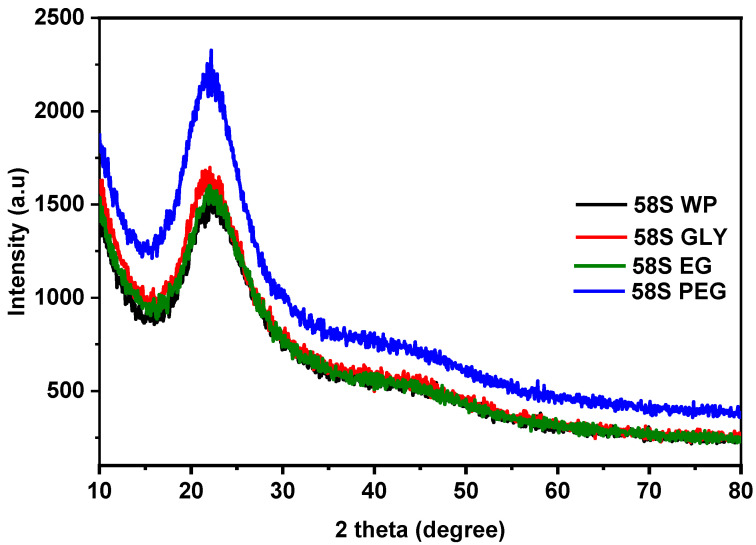
XRD patterns of 58S WP, 58S GLY, 58S EG, and 58S PEG powders dry at 160 °C.

Figure 2 shows the XRD patterns of 58S WP, 58S GLY, 58S EG, and 58S PEG powder samples obtained after drying the sols at 160 °C for 1 h. Only broad diffraction bands centered around 22° (2θ) were observed, suggesting that all powders were amorphous. The addition of polyols to the 58S sol did not affect the amorphous nature or the appearance of secondary silicate phases [51]. 

### 3.3. FT-IR Analysis

**Figure 3:**
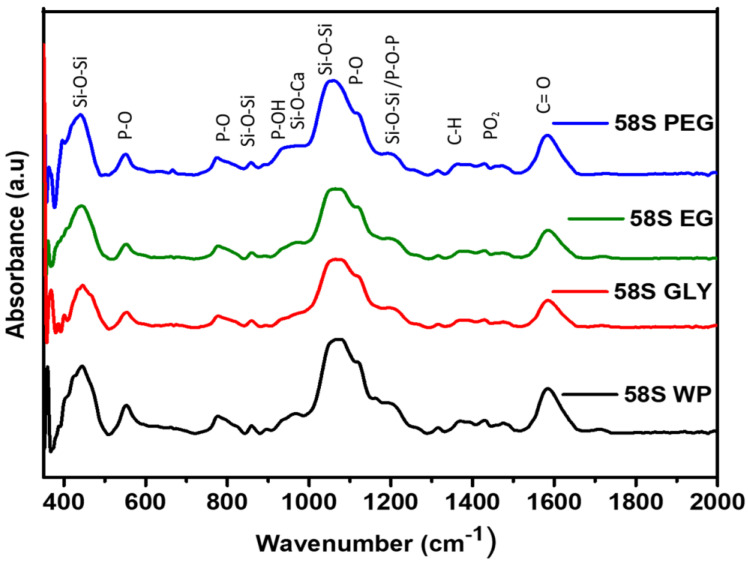
FT-IR spectra of 58S WP, 58S GLY, 58S EG, and 58S PEG sols heat-treated at 160 °C/1 h.

The powder samples used for the XRD analysis were also analyzed by FTIR to demonstrate the formation of the 58S glass structure and to study the functional groups in the glass (Figure 3). A broad band at 450 cm^−1^ associated with the rocking motion perpendicular to the Si-O-Si plane, together with two wider small peaks located around 770 and 850 cm^−1^, related to the symmetric vibrations of PO and the bending of the Si-O-Si band were observed. The widest and most intense band around 900–1300 cm^−1^ is composed of the overlap of the most important bands for this composition. The P-OH (940 cm^−1^) stretching, and Si-O-Ca (960 cm^−1^) vibration modes were identified in the broad band between 920 and 990 cm^−1^, together with the rest of the vibration modes of non-condensed Si-OH. The peaks identified at 1040 and 1190 cm^−1^ can be attributed to Si-O-Si asymmetric and symmetric stretching and P-O-P stretching. The shoulder band at 1230 cm^−1^ corresponds to the symmetric and antisymmetric modes of calcium atoms linked to silica as Si-O-Ca [52]. Between 1330 and 1500 cm^−1^ a very broad and poorly defined peak was observed, where the –CH group of the lactate group overlaps the vibration modes of non-bridging PO_2_ in PO_4_^3−^ (1448 cm^−1^). Their low intensity was related to the low content of phosphates in the composition of the bioactive glass. On the other hand, a band associated with the carboxylic group (1580 cm^−1^) of the lactate group appeared. Considering that lactate is a component of human metabolism, it is physiologically degradable and its presence in the coatings does not represent any problem in terms of biocompatibility. In the region of 2500–3500 cm^−1^ (not shown), bands associated with O-H stretching of adsorbed water and the Si-OH and P-OH groups [47] could be identified.

### 3.4. Contact Angle Measurements

**Figure 4:**
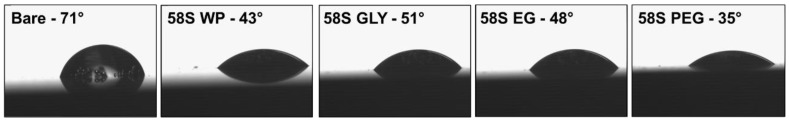
Drops of Hank´s solution on AZ31B alloy with and without 58S coatings during the contact angle measurements.

The wettability of bioactive materials is an important parameter related to bioactivity and cell adhesion. The contact angle of AZ31B substrates with and without coatings was measured by dropping 40 µL of Hank’s solution on the surface (Figure 4). The contact angle on the bare substrate was 71°: hydrogen bubble generation was also observed indicating rapid reactivity and degradation of the Mg alloy. The deposition of a 58S coating decreased the contact angle to around 50° for 58S GLY and 58S EG, 40° for 58S WP, and 35° for 58S PEG. The result was associated with the presence of silanol groups on the surface. No hydrogen bubbles were observed, suggesting at least partial or even complete elimination of degradation of the Mg alloy. For biodegradable implants, a highly hydrophilic surface is preferred over a hydrophobic one because it is more capable of interacting with cells, ions in bodily fluids, and with other biological entities [53,54]. A hydrophobic surface can minimize these interactions. The highest hydrophilicity was observed for the 58S PEG coating.

### 3.5. Hydrogen Evolution and pH Evaluation 

**Figure 5:**
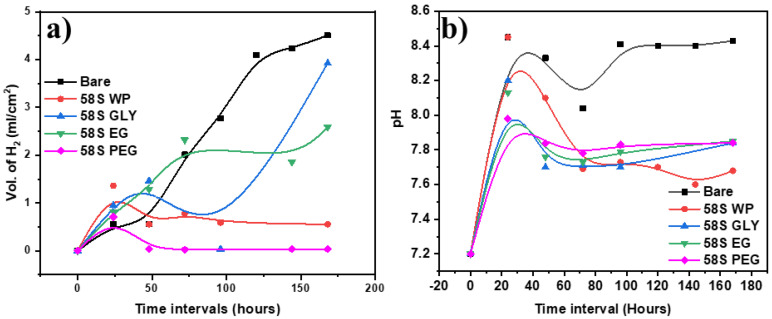
The results of hydrogen gas (H_2_) evolution test. (**a**) pH variation for uncoated and (**b**) coated AZ31B alloys using different polyols as a function of immersion time in Hank´s solution at 37 °C.

The hydrogen gas evolution for the uncoated and coated AZ31B alloys with 58S-based coatings obtained with the use of various polyols was measured in Hank’s solution at 37 °C as a function of immersion time (up to 7 days or 168 h) to evaluate the in vitro biodegradability and to obtain information about the corrosion resistance of the coating systems (Figure 5a).

Spontaneous corrosion of Mg substrate takes place as a result of its reaction in an aqueous environment according to the reaction.

Mg(s) + 2H_2_O → Mg^2+^ +2OH^−^ +H_2_ (g)),

so, the amount of released H_2_ gas is proportional to the degradation rate of the alloy. The higher the H_2_ gas emission, the stronger the alloy degradation. The bare alloy showed the highest evolution of hydrogen, with a maximum release of 4.5 mL/cm^2^ H_2_ gas after 160 h of immersion (Figure 5a). Some irregularities in hydrogen evolution were observed in all AZ31B coated samples, which was associated with the formation of the corrosion products that adhere to the corroded surface and then peel off. In the case of 58S GLY, the H_2_ gas release was unstable: after an initial increase a decrease until 100 h was observed, followed by a marked increase up to a maximum value of 3.9 mL/cm^2^ after 168 h of immersion. The coating 58S EG showed a linear release of H_2_ gas after 75 h of immersion, followed by a constant release rate, reaching a maximum of 2.5 mL/cm^2^. The coatings 58S WP and 58S PEG displayed the most favorable H_2_ gas release behavior. During the first 24 h of immersion in SBF, the volume of H_2_ increased slowly and then decreased until reaching a stable stationary phase, without significant variations, until the end of the test (168 h). 

Similar to H_2_ evolution, the pH change was also monitored with the immersion time. An abrupt pH increase results in a local alkalization of the area causing poisoning and cell death. In the present study, all coatings, including bare AZ31B, were immersed in Hank’s solution for 7 days at 37 °C, as shown in Figure 5b. Initially, a quick increase of pH from 7.2 to 8.4 during the first 24 h was observed for all the samples. However, for bare AZ31B, the pH was maintained at 8.4 for the remaining 7 days while the pH of coated samples decreased, achieving a steady value of 7.8–7.6. The lowest pH was measured for the 58S WP coating. This increment was related to the release of Mg^2+^, Ca^2+^, and HPO_4_^2+^ ions from the substrate and/or coating to the medium. 

In general, the coated samples provide better control of H_2_ and pH evolution than the bare sample. In this case, after 24 h of immersion in Hank´s solution, the 58S coatings could react with the medium, forming an apatite layer that would regulate the variation of pH and favor tissue regeneration. Ng et al. reported that the magnesium degradation rate was considerably higher at the pH values between 7.4 and 5.5, and the alloy was far more stable between 7.4 and 8 [55]. After 20 hours of immersion, the coated samples maintained a pH of 7.7–8, which favored the deposition of the apatite layer.

### 3.6. Immersion Studies in Hank’s Solution

**Figure 6:**
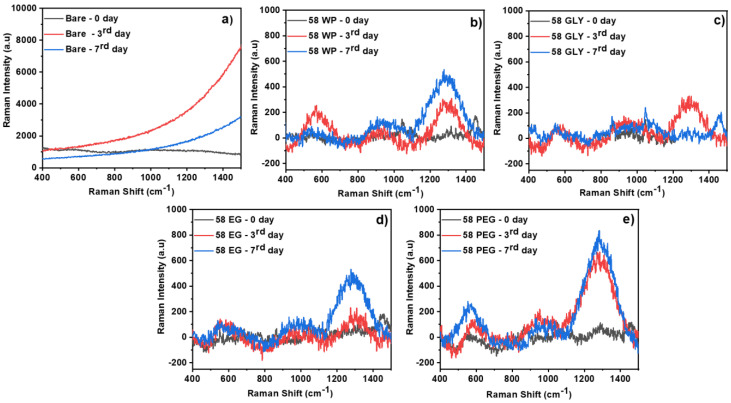
Raman spectra of AZ31B alloys (**a**), 58S WP coating (**b**), 58S GLY coating (**c**), 58S EG coating (**d**), and 58S PEG coating (**e**) maintained in Hank’s solution for 3 and 7 days.

To analyze the formation of the apatite layer on the 58S coatings and uncoated samples, AZ31B and 58S coated with and without polyol were immersed in Hank´s solution at 37 °C for 0, 3, and 7 days and the surface morphology was analyzed by Raman spectroscopy (Figure 6). In the bare sample, the formation of apatite was not confirmed, which was associated with the fast degradation rate of the Mg alloy. In HAP crystals, the characteristic vibration bands of PO_4_ groups are (i) ν2 bending of P-O-P at 472 cm^−1^, (ii) ν4 bending of P-O-P at 563 and 602 cm^−1^, (iii) ν1 stretching of P-O at 960–962 cm^−1^ [56], and (iv) ν2 asymmetrical stretching of (PO_2_) at 1200–1300 cm^−1^[57]. The most noticeable change in amorphous calcium phosphate (ACP) is a 10 cm^−1^ shift of the ν1 stretching towards 950 cm^−1^. As a result, the ACP to HAP transformation is characterized by a shift in the ν1 symmetrical band from a broad peak at about 950 cm^−1^ to a narrow peak around 960 cm^−1^. For the 58S WP coating, the peaks attributed to apatite (ν4 P-O-P and ν1 P-O) and ν2 asymmetrical stretching of PO_2_ (ν_as_ PO_2_) 1250–1300 cm^−1^were observed after 3 and 7 days of immersion. The ν_as_ PO_2_ peaks were more intense after 7 days. In the case of 58S GLY coating, phosphate peaks were also identified, but with lower intensity. In addition, after 7 days of immersion, the intensity of the apatite peak remained unchanged, and the ν_as_ PO_2_ peak decreased. For 58S EG coating, the intensity of apatite and ν_as_ PO_2_ peaks increased gradually with the immersion time. The 58S PEG coating displayed the highest intensities of apatite and ν_as_ PO_2_ peaks of all coated samples during the whole duration of the experiment. 

Considering the H_2_ gas evolution, the 58S GLY coating demonstrated the worst behavior associated with low apatite formation (Figure 6c) after being exposed to Hank’s solution for a longer period. Then again, 58S PEG coating showed the most favorable properties in terms of H_2_ and pH evolution as well as abundant apatite formation. This may be related to the presence of polyethylene glycol, which may have had an impact on the crosslinking of the silica network. A similar study by Li et al. reported that an increase in PEG (molecular weight 600) to silica ratio in the sol promoted the apatite formation [58]. Based on the result the 58S PEG coating was subjected to additional degradation analysis.

### 3.7. Degradation Analysis 

**Figure 7:**
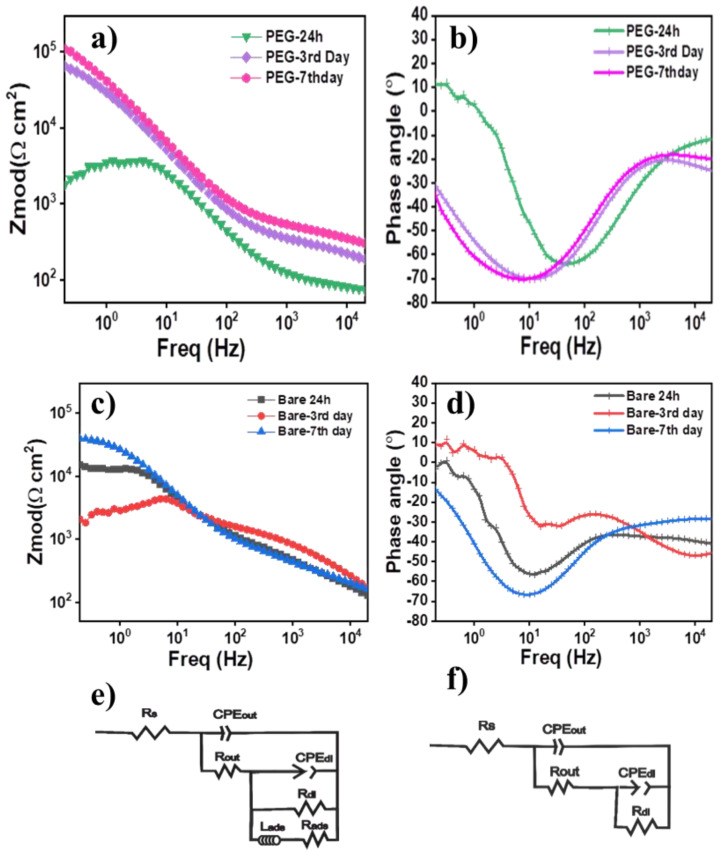
Bode and phase angle plots of (**a**) and (**c**) bare AZ31B alloys, (**b**) and (**d**) 58S PEG coatings immersed in Hank's solution from 24 h to 7 days respectively. Equivalent circuits for (**e**) 7th day Bare AZ31B and (**f**) for 7th day 58S PEG samples.

Electrochemical impedance spectroscopy (EIS) analysis was used to analyze the biodegradation of the uncoated and coated samples during immersion in Hank´s solution at 37 °C. The study was conducted for a 58S PEG-coated sample and AZ31B bare Mg alloy after 24 h, 3 and 7 days of immersion. Figure 7a,b shows the Bode and the phase angle diagrams of both samples as a function of immersion time. The shape of the impedance spectra and the value of the impedance modulus │Z│ at the low-frequency domain (f < 1 Hz) can be directly used to estimate the corrosion resistance performance of the coating [59]. Figure 7a shows the │Z│ modulus (f < 1 Hz) of the bare Mg alloy after 24 h of immersion with a value of ~10^4^ Ω cm^2^. After 3 days of immersion │Z│ modulus decreased down to ~10^3^ Ω cm^2^, one order of magnitude lower than after 24 h. However, after 7 days of immersion, the impedance value increased again above 10^4^ Ω cm^2^. To explain this behavior, different electrochemical phenomena must be considered at the solution/substrate interface. Due to a non-controlled degradation of the bare Mg alloy in Hank’s solution, the apatite layer was unable to form on the surface after 3 and 7 days, as documented by the absence of apatite peaks in the Raman spectra (Figure 7a). The increment of impedance after 7 days of immersion is thus associated with the formation and deposition of an unstable layer composed of corrosion products, such as Mg·(OH)_2_ on the surface, which cannot provide a stable barrier and cannot control the hydrogen or pH evolution during the immersion.

When the 58S PEG coating was deposited on the AZ31B alloy, the Bode plot (Figure 7b) exhibited an impedance modulus of 10^3^ Ω cm^2^ with an inductive behavior at low frequency after 24 h of immersion. However, after 3 and 7 days of immersion, the low frequency inductive loop disappeared, and a capacitive behavior appeared along with an increment of the impedance modulus to the values of about 10^5^ Ω cm^2^. Comparing the impedance behavior of AZ31B bare Mg alloy and 58S PEG coating, the increment of the impedance modulus with the immersion time for the 58S PEG sample could be associated with the formation of stable corrosion products such as apatite documented by Raman spectroscopy (Figure 6e). 

In order to comprehend the corrosion progress of 58S PEG coating with immersion time, the EIS data was fitted using two different equivalent electrical circuit (EEC) as shown in Figure 7c,d. The circuits are constituted of the R_s_ (the solution resistance), the resistance and the constant phase element of the outer layer (R_out_ and CPE_out_), the charge transfer resistance and constant phase element of the Faradaic reaction (R_dl_ and CPE_dl_) on the substrate interface, and the inductive behavior in a low-frequency domain (f < 1 Hz) (R_ads_ and L_ads_). To estimate the total corrosion resistance for each system, the polarization resistance (R_p_) values were calculated according to the fitted equivalent circuit, and R_p_ values are summarized in Table 2. 

The presence of an inductive loop after 24 h of immersion suggests the release of adsorbed ions [60], likely associated with the coating dissolution. The coating dissolution is dominated by the release of Ca^2+^ ions and thus, by the ion exchange between the 58S PEG coating and Hank´s solution [61]. After 3 days, and due to the release of Ca^2+^ ions to the medium, nucleation sites are created on the surface of the sample, and the deposition of apatite on the substrate takes place. The large increment of R_p_ value from 58.81 Ω cm^2^ to 103,055.6 Ω cm^2^ confirms that the corrosion resistance of the substrate was significantly enhanced due to the protective effect of the layer formed because of the precipitation of corrosion products. After 7 days, the R_p_ value increased up to 172,157.4 Ω cm^2^. The slight increment of the Rp value was attributed to the saturation of the nucleation sites and the reduction of ion exchange activity substrate/medium. Although the precipitation rate of the corrosion products seems to be slower, the high Rp value, above 10^5^ Ω cm^2^ after 7 days, confirmed the effectiveness of the 58S PEG coating in terms of control of degradation of the AZ31B substrate in Hank´s solution and confirmed the results of the measurements of hydrogen and pH evolution. 

In conclusion, the in vitro bioactivity of the 58S PEG coating has been demonstrated. It should be noted that the amorphous bioactive materials showed higher bioactivity under physiological conditions than the crystalline ones [62]. The surface biomineralization has been induced and the degradation rate of the AZ31B Mg alloys was been reduced. The developed 58S PEG coating could be considered for potential application in degradable implants in biomedicine.

## 4. Conclusions

Three distinct polyols were incorporated into 58S sol, which was then dip-coated onto an AZ31B alloy substrate. The uniformity and particle homogeneity of the coatings were evaluated, as well as their structural and surface properties and bioactivity. The amorphous nature of 58S sols with and without added polyols was confirmed by structural analysis. Particle size, as measured by scanning electron microscopy (SEM), varies from 0.5 µm ± 0.03 for 58S WP to 0.3 µm ± 0.04 for 58S EG and from 0.1 µm ± 0.08 for 58S GLY to 4.6 µm ± 0.08 for 58S PEG, depending on the polyol used. The presence of elements linked to the composition of the corresponding 58S sol was confirmed by the chemical composition analysis (EDAX). The hydrophilicity of the contact angle decreased from 71° for bare alloy to 35° for 58S PEG coatings under physiological conditions. The linear H_2_ evolution and pH instability of uncoated magnesium alloys with a maximum release of 4.5 mL/cm^2^ and a pH of 8.4 were effectively controlled by 58S PEG coatings to an almost negligible amount of H_2_ evolution and a pH of 7.8 upon 160 h of immersion. Due to the high bioactivity and high degree of crosslinking in the silica network, the PEG-based 58S coating demonstrated the best hydrophilicity, hydrogen evolution, and pH stability during immersion tests, which are essential prerequisites for biomedical applications. The observed control of the surface activity of the 58S PEG coating was attributed to the passivation of the surface through the formation of an apatite layer after 48 h, which was confirmed by the presence of various phosphate peaks in the Raman spectroscopy. The electrochemical impendence study of the degradability of the bare alloy and 58S PEG coating revealed that the 58S PEG coating could control the rapid degradation of Mg alloys under physiological conditions.

Further *in vitro* and in vivo studies are planned to analyze the efficiency of the 58S PEG coating on the degradability of magnesium alloys.

## Figures and Tables

**Table 1:** Aging time of the different 58S sols and thickness of 58S coatings on glass-slide prepared with and without polyols.

58 S Sol	Aging Time (Days)	Coating Thickness (µm)
58 WP	4	1.53 ± 0.01
58 GLY	4	1.65 ± 0.008
58 EG	5	1.5 ± 0.01
58 PEG	3	1.76 ± 0.01

**Table 2:** Fitted values of the equivalent circuit for 58S_PEG samples as a function of immersion time in Hank’s solution.

Sample	R_out_ Ω cm^2^	R_dl_ Ω cm^2^	CPE_out_ Ω^−1^ cm^−2^ s^αcoat^	^αcoat^	R_S_ Ω cm^2^	R_ads_ Ω cm^2^	L_ads_ H cm^2^	R_P_ Ω cm^2^
58S PEG 7th day	527.4 ± 11.4	171,630.00 ± 3846	2.12 × 10^−6^ ± 1.27 × 10^−7^	0.58	40			172,157.4
58S PEG 3th day	352.6 ± 5.95	102,730.00 ± 1303.4	3.88 × 10^−6^ ± 2.6 × 10^−7^	0.60 ± 5.6 × 10^−3^	40			103,055.6
58S PEG 24 h	58.81 ± 15.8	3744 ± 145.7	1.61 × 10^−6^ ± 4.7 × 10^−6^	0.81 ± 0.22	45	2041 ± 177.8	1783 ± 146.8	58.81

## Data Availability

Data are available upon request.

## References

[1] https://www.Imarcgroup.Com/Bio-Implants-Market8.

[2] Tsakiris V., Tardei C., Clicinschi F.M. (2021). Biodegradable Mg Alloys for Orthopedic Implants—A Review. J. Magnes. Alloy..

[3] Mamidi N., Flores Otero J.F. (2023). Metallic and Carbonaceous Nanoparticles for Dentistry Applications. Curr. Opin. Biomed. Eng..

[4] Mamidi N., García R.G., Martínez J.D.H., Briones C.M., Martínez Ramos A.M., Tamez M.F.L., del Valle B.G., Segura F.J.M. (2022). Recent Advances in Designing Fibrous Biomaterials for the Domain of Biomedical, Clinical, and Environmental Applications. ACS Biomater. Sci. Eng..

[5] García-Estrada P., García-Bon M.A., López-Naranjo E.J., Basaldúa-Pérez D.N., Santos A., Navarro-Partida J. (2021). Polymeric Implants for the Treatment of Intraocular Eye Dieases: Trends in Biodegradable and Non-Biodegradable Materials. Pharmaceutics.

[6] Shekhawat D., Singh A., Bhardwaj A., Patnaik A. A Short Review on Polymer, Metal and Ceramic Based Implant Materials A Short Review on Polymer, Metal and Ceramic Based Implant Materials. Proceedings of the IOP Conference Series: Materials Science and Engineering.

[7] Li C., Guo C., Fitzpatrick V., Ibrahim A., Zwierstra M.J., Hanna P., Lechtig A., Nazarian A., Lin S.J., Kaplan D.L. (2020). Design of Biodegradable, Implantable Devices towards Clinical Translation. Nat. Rev. Mater..

[8] Seuba J., Maire E., Adrien J., Meille S., Deville S. (2021). Mechanical Properties of Unidirectional, Porous Polymer/Ceramic Composites for Biomedical Applications. Open Ceramics.

[9] Naghavi S.A., Lin C., Sun C., Tamaddon M., Basiouny M., Garcia-Souto P., Taylor S., Hua J., Li D., Wang L. (2022). Stress Shielding and Bone Resorption of Press-Fit Polyether–Ether–Ketone (PEEK) Hip Prosthesis: A Sawbone Model Study. Polymers.

[10] Dutta S., Gupta S., Roy M. (2020). Recent Developments in Magnesium Metal–Matrix Composites for Biomedical Applications: A Review. ACS Biomater Sci. Eng..

[11] Chalisgaonkar R. (2020). Insight in Applications, Manufacturing and Corrosion Behaviour of Magnesium and Its Alloys—A Review. Mater Today Proc..

[12] Vennimalai Rajan A., Mathalai Sundaram C., Vembathu Rajesh A. (2020). Mechanical and Morphological Investigation of Bio-Degradable Magnesium AZ31 Alloy for an Orthopedic Application. Mater Today Proc..

[13] Zemková M., Minárik P., Jablonská E., Veselý J., Bohlen J., Kubásek J., Lipov J., Ruml T., Havlas V., Král R. (2022). Concurrence of High Corrosion Resistance and Strength with Excellent Ductility in Ultrafine-Grained Mg-3Y Alloy. Materials.

[14] Chandra G., Pandey A. (2020). Biodegradable Bone Implants in Orthopedic Applications: A Review. Biocybern Biomed. Eng..

[15] Shuai C., Li S., Peng S., Feng P., Lai Y., Gao C. (2019). Biodegradable Metallic Bone Implants. Mater. Chem. Front..

[16] Kulinich S., Kuchmizhak A., Honda M., Svetlichnyi V.A., Akimchenko I.O., Rutkowski S., Tran T.-H., Dubinenko G.E., Petrov V.I., Kozelskaya A.I. (2022). Polyether Ether Ketone Coated with Ultra-Thin Films of Titanium Oxide and Zirconium Oxide Fabricated by DC Magnetron Sputtering for Biomedical Application. Materials.

[17] Jia B., Yang H., Han Y., Zhang Z., Qu X., Zhuang Y., Wu Q., Zheng Y., Dai K. (2020). In Vitro and in Vivo Studies of Zn-Mn Biodegradable Metals Designed for Orthopedic Applications. Acta Biomater.

[18] Wang J.-L., Xu J.-K., Hopkins C., Chow D.H.-K., Qin L. (2020). Biodegradable Magnesium-Based Implants in Orthopedics—A General Review and Perspectives. Adv. Sci..

[19] Li H., Wang P., Lin G., Huang J. (2021). The Role of Rare Earth Elements in Biodegradable Metals: A Review. Acta Biomater.

[20] Lin X., Saijilafu, Wu X., Wu K., Chen J., Tan L., Witte F., Yang H., Mantovani D., Zhou H. (2022). Biodegradable Mg-Based Alloys: Biological Implications and Restorative Opportunities. Int. Mater. Rev..

[21] Saberi A., Bakhsheshi-Rad H.R., Abazari S., Ismail A.F., Sharif S., Ramakrishna S., Daroonparvar M., Berto F. (2021). A Comprehensive Review on Surface Modifications of Biodegradable Magnesium-Based Implant Alloy: Polymer Coatings Opportunities and Challenges. Coatings.

[22] Barzegari M., Mei D., Lamaka S.V., Geris L. (2021). Computational Modeling of Degradation Process of Biodegradable Magnesium Biomaterials. Corros. Sci..

[23] Hassan H.W., Grasso V., Korostynska O., Khan H., Jose J., Mirtaheri P. (2021). An Overview of Assessment Tools for Determination of Biological Magnesium Implant Degradation. Med. Eng. Phys..

[24] Hassan H.W., Rahmati M., Barrantes A., Haugen H.J., Mirtaheri P. (2022). In Vitro Monitoring of Magnesium-Based Implants Degradation by Surface Analysis and Optical Spectroscopy. Int. J. Mol. Sci..

[25] Kumar R., Katyal P. (2022). Effects of Alloying Elements on Performance of Biodegradable Magnesium Alloy. Mater. Today. Proc..

[26] Chandra G., Pandey A. (2022). Preparation Strategies for Mg-Alloys for Biodegradable Orthopaedic Implants and Other Biomedical Applications: A Review. IRBM.

[27] Zaffora A., di Franco F., Virtù D., Carfì Pavia F., Ghersi G., Virtanen S., Santamaria M. (2021). Tuning of the Mg Alloy AZ31 Anodizing Process for Biodegradable Implants. ACS Appl. Mater. Interfaces.

[28] Yin Z.-Z., Qi W.-C., Zeng R.-C., Chen X.-B., Gu C.-D., Guan S.-K., Zheng Y.-F. (2020). Advances in Coatings on Biodegradable Magnesium Alloys. J. Magnes. Alloy..

[29] Simchen F., Sieber M., Kopp A., Lampke T. (2020). Introduction to Plasma Electrolytic Oxidation-an Overview of the Process and Applications. Coatings.

[30] Merino E., Durán A., Ceré S., Castro Y. (2022). Hybrid Epoxy-Alkyl Sol–Gel Coatings Reinforced with SiO_2_ Nanoparticles for Corrosion Protection of Anodized AZ31B Mg Alloy. Gels.

[31] Merino E., Durán A., Castro Y. (2021). Integrated Corrosion-Resistant System for AZ31B Mg Alloy via Plasma Electrolytic Oxidation (PEO) and Sol-Gel Processes. Nternational. J. Appl. Glass Sci..

[32] Merino E., Durán A., Castro Y. (2020). The Role of Silane Sol-Gel Coatings on the Corrosion Protection of Magnesium Alloys.

[33] Johari N.A., Alias J., Zanurin A., Mohamed N.S., Alang N.A., Zain M.Z.M. (2022). Anti-Corrosive Coatings of Magnesium: A Review. Mater Today Proc..

[34] Zhang D., Peng F., Qiu J., Tan J., Zhang X., Chen S., Qian S., Liu X. (2021). Regulating Corrosion Reactions to Enhance the Anti-Corrosion and Self-Healing Abilities of PEO Coating on Magnesium. Corros. Sci..

[35] Omar S.A., Ballarre J., Castro Y., Martinez Campos E., Schreiner W., Durán A., Cere S.M. (2020). 58S and 68S Sol-Gel Glass-like Bioactive Coatings for Enhancing the Implant Performance of AZ91D Magnesium Alloy. Surf. Coat. Technol..

[36] Saji V.S. (2021). Electrophoretic (EPD) Coatings for Magnesium Alloys. J. Ind. Eng. Chem..

[37] Deshmukh K., Kovářík T., Křenek T., Docheva D., Stich T., Pola J. (2020). Recent Advances and Future Perspectives of Sol–Gel Derived Porous Bioactive Glasses: A Review. RSC Adv..

[38] Vallet-Regi M., Salinas A.J. (2021). Mesoporous Bioactive Glasses for Regenerative Medicine. Mater. Today Bio..

[39] Müller V., Jobbagy M., Djurado E. (2021). Coupling Sol-Gel with Electrospray Deposition: Towards Nanotextured Bioactive Glass Coatings. J. Eur. Ceram. Soc..

[40] Omar S., Pastore J., Bouchet A., Pellice S., Ballarín V., Cere S., Ballarre J. (2016). SiO_2_-CaO-P_2_O_5_ (58S) Sol Gel Glass Applied onto Surgical Grade Stainless Steel by Spray Technique: Morphological Characterization by Digital Image Processing. Biomed. Glasses.

[41] Islam S. (2021). Impact of PH on Structural and Sensing Characteristics of Cresol Red Encapsulated Polyethylene Glycol Assisted Silica Nanomatrix: Sol-Gel Method. Opt. Mater. (Amst).

[42] Ghamarpoor R., Jamshidi M. (2022). Synthesis of Vinyl-Based Silica Nanoparticles by Sol–Gel Method and Their Influences on Network Microstructure and Dynamic Mechanical Properties of Nitrile Rubber Nanocomposites. Sci. Rep..

[43] Ravaine D., Seminel A., Vincens M. (1986). A new family of organically modified silicates prepared from gels. J. Non-Cryst. Solids.

[44] Giz A.S., Aydelik-Ayazoglu S., Catalgil-Giz H., Bayraktar H., Alaca B.E. (2019). Stress Relaxation and Humidity Dependence in Sodium Alginate-Glycerol Films. J. Mech. Behav. Biomed. Mater..

[45] Stefanescu M., Stoia M., Stefanescu O. (2007). Thermal and FT-IR Study of the Hybrid Ethylene-Glycol-Silica Matrix. J. Solgel. Sci. Technol..

[46] Catauro M., Bollino F., Papale F., Ferrara C., Mustarelli P. (2015). Silica-Polyethylene Glycol Hybrids Synthesized by Sol-Gel: Biocompatibility Improvement of Titanium Implants by Coating. Mater. Sci. Eng. C.

[47] Lee J.H., Lee H.B., Andrade J.D. (1995). Blood Compatibility of Polyethylene Oxide Surfaces. Prog. Polym. Sci..

[48] Alcantar N.A., Aydil E.S., Israelachvili J.N. (2000). Polyethylene Glycol-Coated Biocompatible Surfaces. J. Biomed. Mater. Res..

[49] Song G., Atrens A., StJohn D., Mathaudhu S.N., Luo A.A., Neelameggham N.R., Nyberg E.A., Sillekens W.H. (2016). An Hydrogen Evolution Method for the Estimation of the Corrosion Rate of Magnesium Alloys. Essential Readings in Magnesium Technology.

[50] Agustín-Sáenz C., Sánchez-García J.Á., Machado M., Brizuela M., Zubillaga O., Tercjak A. (2018). Broadband Antireflective Coating Stack Based on Mesoporous Silica by Acid-Catalyzed Sol-Gel Method for Concentrated Photovoltaic Application. Sol. Energy Mater. Sol. Cells.

[51] Chen J., Zeng L., Chen X., Liao T., Zheng J. (2018). Preparation and Characterization of Bioactive Glass Tablets and Evaluation of Bioactivity and Cytotoxicity in Vitro. Bioact. Mater..

[52] Bui X.V., Dang T.H. (2019). Bioactive Glass 58S Prepared Using an Innovation Sol-Gel Process. Process. Appl. Ceram..

[53] Liu Z., Liu X., Ramakrishna S. (2021). Surface Engineering of Biomaterials in Orthopedic and Dental Implants: Strategies to Improve Osteointegration, Bacteriostatic and Bactericidal Activities. Biotechnol. J..

[54] Almassri H.N.S., Ma Y., Dan Z., Ting Z., Cheng Y., Wu X. (2020). Implant Stability and Survival Rates of a Hydrophilic versus a Conventional Sandblasted, Acid-Etched Implant Surface: Systematic Review and Meta-Analysis. J. Am. Dent. Assoc..

[55] Ng W.F., Chiu K.Y., Cheng F.T. (2010). Effect of PH on the in Vitro Corrosion Rate of Magnesium Degradable Implant Material. Mater. Sci. Eng. C.

[56] Stammeier J.A., Purgstaller B., Hippler D., Mavromatis V., Dietzel M. (2018). In-Situ Raman Spectroscopy of Amorphous Calcium Phosphate to Crystalline Hydroxyapatite Transformation. MethodsX.

[57] Hussin R., Shawal Husin M., Nur Fazliana Abdul Halim D., Hamdan S. (2010). Vibrational studies of crystalline phase strontium magnesium phosphates doped with Eu_2_O_3_. Solid State Sci. Technol..

[58] Li A., Shen H., Ren H., Wang C., Wu D., Martin R.A., Qiu D. (2015). Bioactive Organic/Inorganic Hybrids with Improved Mechanical Performance. J. Mater. Chem. B.

[59] Durán K.S., Hernández N., Rueda L.M., Hernández-Barrios C.A., Coy A.E., Viejo F. (2021). Design of Multilayer Hybrid Sol-Gel Coatings with Bifunctional Barrier-Bioactive Response on the Elektron 21 Magnesium Alloy for Biomedical Applications. J. Magnes. Alloy..

[60] Su Y., Niu L., Lu Y., Lian J., Li G. (2013). Preparation and Corrosion Behavior of Calcium Phosphate and Hydroxyapatite Conversion Coatings on AM60 Magnesium Alloy. J. Electrochem. Soc..

[61] Zhang Q., Chen J., Feng J., Cao Y., Deng C., Zhang X. (2003). Dissolution and Mineralization Behaviors of HA Coatings. Biomaterials.

[62] Lombardi M., Gremillard L., Chevalier J., Lefebvre L., Cacciotti I., Bianco A., Montanaro L. (2013). A Comparative Study between Melt-Derived and Sol-Gel Synthesized 45S5 Bioactive Glasses. Key Eng. Mater..

